# Will Costliness Amplify the Signalling Strength of Past Pro-Environmental Behaviour? Exploratory Study on Autonomy

**DOI:** 10.3390/ijerph181910216

**Published:** 2021-09-28

**Authors:** Shizhen Bai, Yan Wang, Shengxiang She, Sheng Wei

**Affiliations:** 1 School of Management, Harbin University of Commerce, Harbin 150028, China; baishzh@hrbcu.edu.cn (S.B.); weisheng@hrbcu.edu.cn (S.W.); 2 School of Business and Administration, Guizhou University of Finance and Economics, Guiyang 550025, China; yangwanggc@163.com

**Keywords:** pro-environmental behaviour (PEB), environmental self-identity (ESI), costliness, autonomy, signalling strength

## Abstract

Research has shown that the extent to which previous environmental actions are linked to people’s environmental self-identity influences subsequent environmentally-friendly behaviour. The study empirically examined the influences of recycling efforts on subsequent pro-environmental behaviour by PLS (partial least squares) structural equation modelling based on the survey data of 426 respondents in China. The results indicate that recycling efforts have a positive effect on pro-environmental behaviour through the mechanism of feelings of pride and environmental self-identity. We hypothesise that past pro-environmental behaviour is more likely to promote an individual’s environmental self-identity when the behaviour is incurred with a higher costliness. However, the results show that only when individuals autonomously perform costly recycling behaviour, the signalling strength of previous recycling efforts is higher to promote environmental self-identity. On the contrary, the high costliness weakens the signalling strength of previous recycling efforts through producing negative emotions. Our results show that when reminding people of their past pro-environmental behaviour in order to promote future pro-environmental behaviour, it is useful to emphasize the autonomously taken costliness of behaviour as it can strongly signal that one is a pro-environmental person, thus as to strengthen environmental self-identity.

## 1. Introduction

Many people are used to taking some kind of pro-environmental behaviour (PEB), also named as environmental responsibility behaviour, environmentally friendly behaviour, environmental sustainable behaviour [1], refers to the behaviour that is beneficial or can reduce the damage to the environment to the full, such as putting glass containers in recycling bins or collecting plastic bags for cleaners to take away. However, will such action also increase the possibility of individuals making other PEBs later? For example, if a person usually has the good habit of recycling waste, when he or she is asked to donate for environmental protection, will he or she be more inclined to donate, or will he or she choose not to donate because he or she feels that he or she has made efforts?

Many studies have shown that people’s past PEBs can promote future PEBs. For example, a study on Chinese consumer behaviour shows that household waste sorting promotes green consumption behaviour [2]. In addition, when people recall the past PEB, they show higher pro-environmental intentions [3]. Reminding people of their past pro-environmental efforts or labelling people as environmentalists can increase their environmentally friendly decisions [4,5,6,7].

Academia has made great progress in understanding the emerging topic of behavioural spillovers in recent years, particularly in the field of PEB. Specifically, PEB spillover is the observable causal impact of a PEB on other related PEBs, in which the occurrence of the initial behaviour is usually subject to some policy or business intervention [8,9].

PEB spillover effect may be positive, that is, the performance of a PEB increases the possibility of another PEB, as in the examples above. However, evidence from other studies may indicate negative spillover. For example, some studies have found that the implementation of recycling behaviour and even the expectation will lead to wasting [10,11] or lower support for green funds [12]. There is also evidence from China that in a community-based field experiment, encouraging households to classify garbage led to a significant increase in household power consumption [13]. In another study based on a Chinese consumer survey, consumers’ usual recycling efforts promote resource consumption [14].

In addition, the study on moral licensing shows that the previous PEB may restrain rather than promote the subsequent PEBs, because once people perform an environmental “good deed,” they will feel that it is reasonable to slack off on other PEBs [15], or believe that they are allowed to commit immoral behaviour in the future. Therefore, in some cases, the implementation of PEB will produce positive spillover (the increase of subsequent PEB or the reinforcement of environmental attitude), while in other cases, it will lead to negative spillover (the decrease of future PEBs and the weakening of environmental attitude).

Some studies try to reveal the determinants of the PEB spillover. The theory of self-perception holds that individuals know themselves by observing the meaning of the behaviour, just as they know others [16]. Therefore, the implementation of PEB may cause individuals to regard themselves as “environmentalists.” In fact, when people are aware of their past environmental behaviour, they may feel a stronger environmental self-identity (ESI) [6,17]. Recent studies have shown that environmental self-identity is stronger when the initial PEB more strongly signals that one is a pro-environmental person. This indicates that the influence of previous PEB on environmental self-identity depends on the signalling strength of the behaviour [7].

van der Werff et al. [7] proposed that three kinds of information about PEB have the potential to affect the signalling strength, one of which is the difficulty of PEB. The harder one tries to take some pro-environmental actions, the more this behaviour can signal the environmental self-identity. In Ma et al. [14] study, positive emotion such as feelings of pride is related to individual’s effort to perform recycling. Therefore, if an individual bears a higher costliness for the past PEB, he or she may produce stronger environmental self-identity as well as positive emotions, resulting in stronger positive spillover effects.

However, does the costliness of PEB necessarily enhance the signalling effect and then cause positive spillover? The evidence is mixed. Actually, a meta-analysis of PEB spillover found that past PEBs with more difficulty decreased positive spillover in terms of intentions and produced no spillover on real behaviour [18]. This study believes that previous studies have ignored the autonomy of PEB, especially costly PEB. Considering those individuals who are forced to perform a costly PEB possibly due to social pressure (such as social norms and peer attitudes), then the higher costliness may result in stronger negative emotions, which may lead to a different story.

Therefore, this study aims to confirm the spillover effect of PEB and the moderating role of behaviour costliness on signalling strength in the context of the Chinese consumer. Further, we try to investigate and reveal the role of individual autonomy in accepting the costliness. Different from previous experimental studies, we chose to survey individuals’ past recycling efforts and then examine the spillover effect on a series of other PEBs. In addition, this study not only verified the mediating role of environmental self-identity but also incorporated a positive emotion into the psychological mechanism. Our study does find that autonomy is a crucial factor affecting the behaviour signalling effect.

### 1.1. Environmental Self-Identity

Many scholars paid attention to why past behaviour would signal one’s identity. Studies have shown that people want to protect their self-image [19] when making choices, thus they would “consult” with their identities and they tend to act according to the norms prescribed by these identities [20]. People may not fully know about their moral type, thus PEB may help them update their views on themselves: if I behaved pro-environmentally, I must be a pro-environmental person, thus I will be more pro-environmental subsequently [21].

Similarly, some recent studies on decision-making in the context of prosocial behaviour incorporated concerns about self-image and identity into the models, indicating that behaviour in conflict with self-identity will reduce the agent’s utility, while behaviour in support of self-identity will increase the utility [22,23]. In the multiple self-model proposed by Bodner and Prelec [24], a probability distribution over possible moral types is used to express the self-image. People attach importance to a secure self-image with the quality of conforming to social norms and personal beliefs. However, individuals are not sure of their real moral type, thus they have to infer their moral type based on their previous behaviour [21,25].

The psychological study also explored how people determine their self-identity from observing their behaviour [16,26]. A classic experimental study by Bem and McConnell [27] showed that individuals developed new attitudes based on inferencing about recent behaviour. In fact, these results showed that people generally tend to think that the present self is similar to the past self and that they are consistent over time [28,29].

van der Werff et al. [7] suggested that the promotion of previous environmental activities on future PEBs depends on the relevance between the initial action and environmental self-identity, which is defined as the extent to which individuals regard themselves as pro-environmental people. Environmental self-identity is a crucial factor in investigating whether an initial PEB will cause positive spillover. In fact, environmental self-identity is proved to predict PEB and pro-environmental attitude, such as purchasing sustainable products, saving energy, reducing waste, turning to sustainable energy, choosing green transportation options, and supporting environmental policies [6,7,30,31,32,33,34].

An alternative explanation is that environmental self-identity is a more underlying influencing factor, which affects both past PEB and future PEB, while past behaviour does not necessarily indicate environmental enhancement of self-identity. However, in the design of this study, the respondents were asked to indicate how often they performed the PEB in the past, we actually manipulated past behaviour by reminding people of their previous pro-environmental actions in a similar way as Cornelissen et al. [8], which can promote environmental self-identity [6,7]. Therefore, the following assumptions were proposed:

**Hypothesis** **1a (H1a).** *PEB positively affects environmental self-identity.*

**Hypothesis** **1b (H1b).** *Environmental self-identity positively affects other PEB.*

### 1.2. Feeling of Pride

Pride is a positive emotion that arises from a specific achievement or prosocial behaviour [35]. The feeling of pride comes from the individual’s evaluation of own behaviour [36,37]. That is, the behaviour is in line with values and morality. On the contrary, when individuals realize that their behaviour is immoral and inappropriate, they may feel ashamed or guilty for themselves [38,39]. These findings are consistent with prior studies [40,41], who found that individuals felt pride when they achieved positive outcomes. As pro-environmental behaviour is positive in moral and socially desired, people would feel that they have achieved progress towards environmental goals when actively engaged in pro-environmental behaviour, thus increasing their pride feelings. Therefore, we put forward the following hypothesis:

**Hypothesis** **2a (H2a).** *PEB positively affects feelings of pride.*

Empirical research shows that pride is positively related to one’s self-concept [14,42]. Therefore, the pride generated by engaging in pro-environmental activities can promote a positive self-concept as an environmentalist. In addition, some studies have found that personal self-evaluation can directly strengthen self-identity. Specifically, people compare their behaviour with related standards, and if they conform to these standards, they will have a good feeling of themselves [43,44]. Therefore, when people believe that it is good to engage in pro-environmental activities, the arising pride can enhance their self-identity in the environment. Based on this reasoning, we propose the following hypothesis:

**Hypothesis** **2b (H2b).** *Feelings of pride from PEB positively affect environmental self-identity.*

### 1.3. Costliness of PEB

Gneezy, Imas, Brown, Nelson and Norton [45] proposed that the costliness is a critical moderator of when previous behaviour leads to either consistency or licensing effects. Basically, any pro-environmental behaviour involves some kind of cost and effort. Cost refers to monetary expenses, such as buying a new energy vehicle. Effort refers to any non-financial expense required to perform a behaviour, such as the time required to recycle garbage [46]. In this paper, we generally use the term of costliness to represent the cost and effort of pro-environmental behaviour.

Since this study focuses on recycling behaviour, it is necessary to discuss the cost of recycling behaviour. According to the U.S. Environmental Protection Agency [47], recycling is the process of collecting and processing materials that would otherwise be thrown away as trash and turning them into new products. In daily life, different recycling behaviour correspond to different difficulties or costliness, which requires individuals to pay a certain degree of effort, such as the distance they walk or the time they spend to recycle specific wastes. Individuals often need to pay corresponding costs, such as time, money, energy and so on.

The costliness of different recycling behaviour is different. For example, when disposing of recyclable garbage, sometimes there is a recycling bin nearby, and sometimes it takes a long distance to find the recycling bin. Many times, individuals need to remove contamination from recyclable materials (such as plastic and glass containers) before recycling [48]. For example, since 2020, Shanghai in China has taken the lead in mandatory waste classification, stipulating that only plastic waste sorted and cleaned can be used as renewable resources. Some hazardous wastes, such as used batteries, need to be placed at specific recycling points, and even consumers need to mail them back to the manufacturer.

In reality, some consumers may not strictly abide by the corresponding recycling specifications considering the recycling costliness. In addition, individuals’ perceptions of the costliness of the same recycling behaviour will also vary. For example, people with plenty of time may think that the process of going out to throw garbage is very easy, and they have to go out for a walk anyway. However, this is time-consuming for busy people. Generally speaking, the recycling costliness is diversified and has individual heterogeneity.

According to the self-perception theory [16], people are more likely to attribute a costly (vs. costless) environment-friendly action to an internal identity. Indeed, the signalling effect of behaviour costliness has been found in the study of moral behaviour. In the economic model of prosocial behaviour, behaviour is the signal of a person’s moral type. Bénabou and Tirole [49] believe that the lower the signal cost, the smaller information contribution to a person’s moral type. In the two classical consistency paradigms of cognitive dissonance and the foot in the door, the costliness of implementing certain moral behaviour is key to produce subsequent moral consistency, although these researchers do not clearly identify costliness as a key mechanism.

Therefore, individuals’ moral identity is more strengthened when they implement a moral action that requires more effort than when they implement a moral action that requires less effort [45]. As more direct evidence, van der Werff et al. [7] found that reminding people of difficult and unique previous PEBs was more helpful to strengthen one’s environmental self-identity. Therefore, when individuals engage in costly PEBs, environmental self-identity is particularly likely to be strengthened.

**Hypothesis** **3 (H3).** *Behavioural costliness positively moderates the spillover effect of PEB.*

**Hypothesis** **3a (H3a).** *Behavioural costliness positively moderates the impact of PEB on environmental self-identity. That is, the higher the costliness, the greater the positive effect of PEB on environmental self-identity.*

**Hypothesis** **3b (H3b).** *Behavioural costliness positively moderates the impact of pro-environmental behaviour on feelings of pride. That is, the higher the costliness, the greater the positive effect of PEB on feelings of pride.*

The above hypothesis includes a precondition of the behaviour costliness, that is, individuals are willing to accept the costliness of PEB. However, the signalling strength of the PEB may also depend on whether or not the behaviour is performed autonomously. Only when individuals freely choose to perform PEBs can the PEBs be attributed to internal factors. In reality, some individuals may not voluntarily accept the cost of certain pro-environmental behaviour. For example, people often have to perform some difficult recycling behaviour due to social pressure. Therefore, negative emotions such as annoyance, unhappiness accompanied by high costliness will weaken the positive impact of PEB on feelings of pride and environmental self-identity.

If a person’s moral self-identity is not strengthened, the previous PEB may lead to the opposite result. According to the literature on moral licensing [50], individuals accumulate credits in a metaphorical moral bank account and later use them to buy out of positive behaviour or offset negative behaviour, retaining an overall positive balance on their moral ledger despite clear withdrawals. Therefore, individuals earn moral credits through performing PEB. When the moral credits accumulate to a certain amount, they will feel qualified to relax the requirements for continued PEBs.

Accordingly, we speculate that for individuals with different autonomy, the costliness will have moderation effects in the opposite direction. Specifically, for those individuals who involuntarily accept the recycling costliness, the recycling costliness will negatively moderate the signalling effect of recycling efforts. For those individuals who voluntarily accept the recycling costliness, the recycling costliness will still positively moderate the signalling effect of recycling efforts.

**Hypothesis** **4 (H4).** *Autonomy will moderate the moderation effect of behavioural costliness on signalling effect of PEB.*

**Figure 1:**
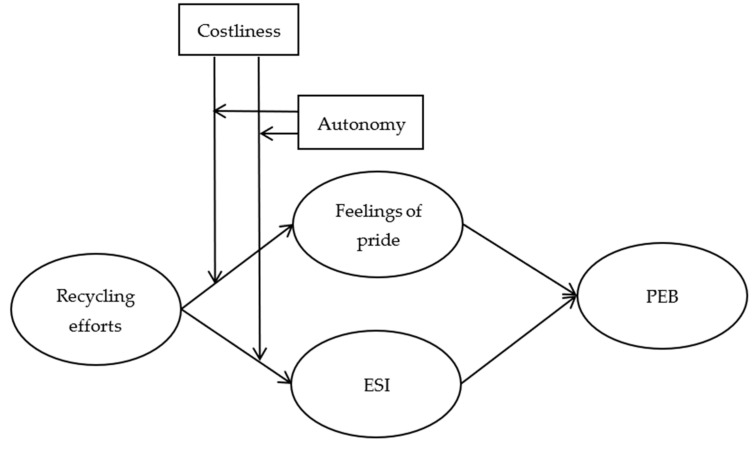
Conceptual model.

To better understand the relationship between recycling efforts and subsequent PEB, we developed a conceptual model (Figure 1).

## 2. Materials and Method

### 2.1. Data Collection and Sample

This research chooses a questionnaire-based survey to measure the respondents’ daily recycling behaviour, which is also a reminder of pro-environmental behaviour. We commissioned a professional survey company to collect data. The target of this research was the individuals who often implemented recycling in the recent period. A total of 500 questionnaires were obtained, of which 426 were effective. According to the gender distribution of the samples, there were 172 men and 254 women. In terms of age, samples under 18 years old accounted for 2%, samples between 18–25 years old accounted for 44%, samples between 26–30 years old accounted for 32%, samples between 31–40 years old accounted for 14%, samples between 41–50 years old accounted for 6% and samples between 51–60 years old accounted for 2%.

### 2.2. Measurement

The questionnaire consisted of 6 constructs, including environmental self-identity, recycling efforts, feelings of pride, the costliness of recycling, pro-environment behaviour and autonomy. The first 5 constructs were measured by existing scales adopted from a prior study (see Appendix A). We adapted them in order to fit the context of the study. As a common practice, the main constructs were measured using a 7-point Likert scale (from 1 = strongly disagree to 7 = strongly agree). Because the items of measurement were translated into the Chinese language from English, back-translations were implemented to ensure semantic accuracy [51]. Recycling efforts indicate the extent to which individuals usually participate in recycling behaviour. Typical items included: I usually separate and dispose of all recyclable materials. However, recycling efforts only measure the degree of individual past recycling behaviour, but it does not capture the costliness of recycling behaviour, nor does it reflect whether individuals voluntarily implement recycling. Therefore, we borrowed the concept of the costliness of recycling from We et al. [52] to reflect the perceived difficulty and cost of individuals involved in recycling behaviour. Typical items included: I spend time on recycling. Since the subjects of this study were Chinese, we used the items from the Chinese general social survey (CGSS) to measure other pro-environmental behaviour, which included 9 items. We developed 7 items to measure autonomy as there were no available scales to adopt. In order to develop appropriate items, we held a focus group discussion and requested the participants: please describe how you feel when you are not voluntarily engaging in recycling. The final measure included 7 items, and the typical items were: I was not willing; I had no choice.

## 3. Results

We used PLS-SEM to analyse the data partly due to the non-normal distribution of the variables in our sample. PLS is suitable because it does not assume normal distributions and allows for analyses with small samples [53]. If applied properly, PLS-SEM can produce better estimations of structural models than covariance-based SEM [54,55]. Using the Chinese version of SmartPLS 3.0, a two-stage analytical procedure was applied to analyse the data [53,56]. In the first stage, the measurement model was assessed in terms of its reliability and validity. In the second stage, the structural model was examined. The significance of the model estimates was based on a bootstrapping procedure with 5000 samples [53].

### 3.1. Reliability and Validity Test

We conducted confirmatory factor analysis (CFA) to evaluate the psychometric adequacy of the constructs for the five adopted measurements. As shown in Table 1, all factor loading was significant (*p* < 0.001), ranging from 0.725 to 0.982. Composite reliability (CR) for the main constructs ranged from 0.872 to 0.976 and Cronbach’s alphas were 0.872 or above (see Table 2), well above the benchmark of 0.7, implying reliable measures. The average variance extracted (AVE) of all constructs ranged from 0.612 to 0.871, exceeding the recommended value of 0.50. The discriminant validity of the constructs was assured as the AVE of each construct exceeded its squared correlation to any other construct.

### 3.2. Hypotheses Testing

(1) SEM

The results based on the structural equation model are shown in Table 3. Recycling efforts had a significant positive influence on environmental self-identity (β = 0.216, t = 3.162, *p* = 0.012). Environmental self-identity had a significant positive influence on pro-environmental behaviour (β = 0.448, t = 5.188, *p* = 0.000). Recycling efforts had a significant positive effect on feelings of pride (β = 0.418, t = 8.625, *p* = 0.000). Feelings of pride had a significant positive impact on environmental self-identity (β = 0.524, t = 9.126, *p* = 0.000). As a result, we argue that the findings were consistent with the predictions by H1 and H2. The standard root-mean-square residual (SRMR) of the structural model was 0.045, which was under the benchmark of 0.08, implying a good absolute goodness-of-fit. Besides, the Normed Fit Index (NFI) was 0.922, above the recommended threshold of 0.9, and the d_ULS and d_G were lower than 0.95 [57].

(2) Test of moderating effect of costliness

**Figure 2:**
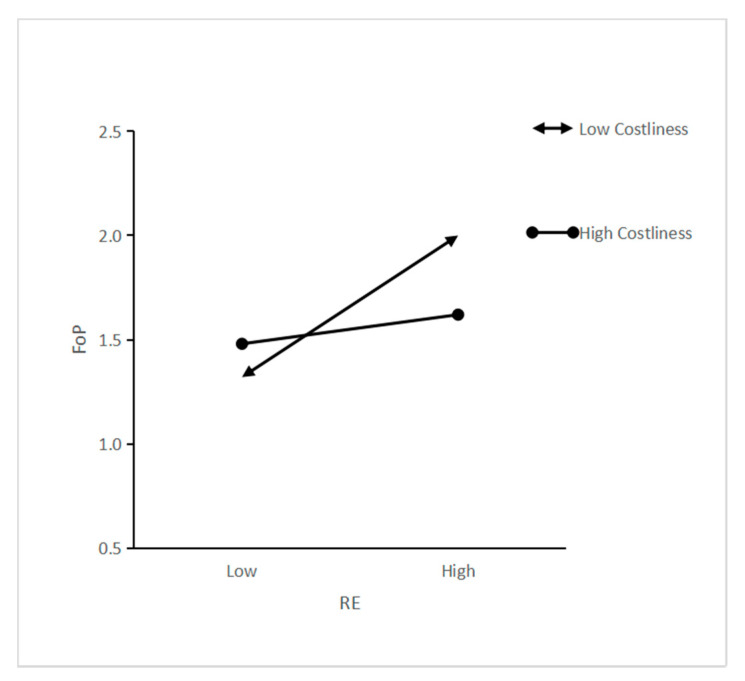
Two-way interaction of recycling effort and costliness.

Hierarchical multiple regression analysis was used to test the moderating roles of costliness on the relationship between recycling efforts and environmental self-identity (and feelings of pride). Results show that the interaction of recycling efforts × costliness had a non-significant negative effect on environmental self-identity (β = −0.08, *p* > 0.05), which rejects H3a. In addition, the interaction of recycling efforts × costliness on feelings of pride was significantly negative (β = −0.160, *p* < 0.001), implying that costliness plays a negative moderating role in the relationship between recycling efforts and feelings of pride, which is contrary with H3b. That is, when costliness was high, the relationship between recycling efforts and feelings of pride was weaker than when costliness was low. The two-way interaction effects are shown in Figure 2.

(3) Test of autonomy of costliness

A three-way interaction term: recycling efforts × costliness × autonomy is incorporated into the regression equation. The results show that the interaction term was significantly associated with feelings of pride (β = 0.156, *p* < 0.01) and environmental self-identity (β = 0.148, *p* < 0.01). It means that with the increase of recycling efforts when individuals perceive higher costs and higher autonomy, the feelings of pride and environmental self-identity were higher. On the contrary, when the autonomy was lower, the higher the cost was accompanied by lower feelings of pride and environmental self-identity.

**Figure 3:**
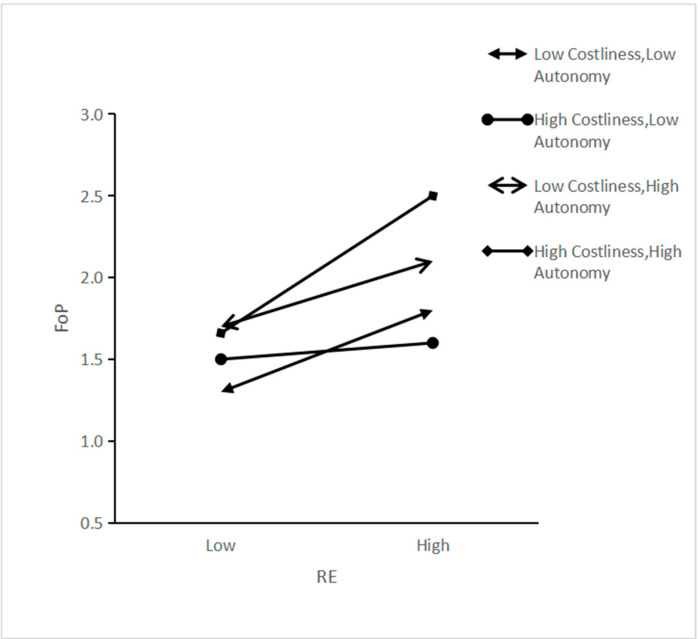
Three-way interaction of recycling effort, costliness and autonomy.

Drawing on Dawson and Richter [58], we divided the costliness and autonomy into four cases: low costliness-low autonomy, low costliness-high autonomy, high costliness-low autonomy and high costliness-high autonomy and draws a three-way interaction effect diagram. Considering that interaction items have a similar effect on feelings of pride and environmental self-identity, we only plotted the three-way interaction effect diagram for feelings of pride. As shown in Figure 3, the slopes of the fitting curves were positive in four cases. However, the slope was the largest in the case of high costliness-high autonomy, indicating that the effect of recycling efforts was the highest when individuals voluntarily took high costliness. Under low autonomy conditions, with the higher the costliness, the effect of recycling efforts on feelings of pride decreases.

To further test the role of autonomy, we divided the samples into two groups according to the level of autonomy. When the average score of the respondents’ autonomy was greater than four, they were allocated to the high autonomy group, N = 196. When the average value was less than or equal to four, the subject was assigned to the low autonomy group, N = 230. Then, the moderating effect of the costliness was tested again for the two groups, respectively.

For the low autonomy group, the interaction of recycling efforts × costliness on feelings of pride was negative and significant (b = −0.31, *p* < 0.001), suggesting a negative moderating effect. Besides, the interaction of recycling efforts × costliness on environmental self-identity was also negative and significant (b = −0.20, *p* < 0.001). For the high autonomy group, the interaction of recycling efforts × costliness on environmental self-identity was positive and significant (b = 0.29, *p* < 0.001), showing a positive moderating effect. Besides, the interaction of recycling efforts × costliness on feelings of pride was also positive and significant (b = 0.20, *p* < 0.001). Based on these results, it was concluded that the autonomy was a further moderating variable to determine the moderating effect of the costliness of recycling. As a result, the H4 was supported.

## 4. Discussion

We tested whether past pro-environmental behaviour could enhance an individual’s environmental self-identity, and how environmental self-identity turns to affect other pro-environmental preferences. Then, we tested under what circumstances the pro-environmental behaviour has a stronger signalling effect and spillover effect. Some studies have shown that if the previous pro-environmental behaviour can enhance environmental self-identity, it will particularly encourage the subsequent PEBs [7]. They believe that past behaviour needs to imply some aspects of the actor to enhance environmental self-identity. The present study proposes that when the initial behaviour involves autonomous acceptance of higher costliness, past PEBs will enhance environmental self-identity to a greater extent because of stronger signalling strength. On the contrary, if someone involuntarily pays a higher costliness in performing PEBs, the signalling effect will be weakened, thus weakening the environmental self-identity.

We do find that individuals’ past recycling efforts have a positive association with environmental self-identity and feelings of pride and spillover to other PEB intentions. We were surprised to find that without considering individual autonomy, the costliness of recycling behaviour negatively moderated the effect of recycling efforts on feelings of pride and environmental self-identity. However, when considering individual autonomy, the truth was brought into the daylight. It was revealed that whether or not an individual voluntarily takes the costliness related to recycling, it was found that it determined the signalling strength of costliness as a moderated moderation effect. Specifically, for the high autonomous group, the higher the recycling costliness, the stronger the environmental self-identity as well as feelings of pride caused by recycling efforts, which means that the recycling cost enhances the signalling effect of previous PEBs. For the low autonomous group, the higher the recycling costliness, the less the past recycling efforts can promote feelings of pride as well as environmental self-identity. We believe that the nonautonomous implementation of costly PEBs will produce negative emotions, which weakens the feelings of pride, and then negatively relates to the signalling effect of recycling efforts on environmental self-identity.

Our research supports Gneezy et al. [45] who explored the crucial role of costliness in emergency of behaviour consistency through experimental methods. Their results confirmed that costly prosocial behaviour was a signal of prosocial identity and positively affects subsequent prosocial behaviour such as truth-telling and buying gifts. Our results also support the findings of van der Werff et al. [7] on the role of initial PEB as a signal of environmental self-identity. However, there were several key differences between this study and previous studies. Firstly, this study focuses on and reveals the key role of autonomy in the signalling effect on identity for the first time, which is also the major contribution of this study. Previous studies found that past PEBs may inhibit or promote subsequent PEBs. The study adds more insights to the inconsistent findings from earlier studies. Our results suggest that environmentally friendly actions are not necessary to promote subsequent PEBs, which may be because the past behaviour did not strongly signal that a person was pro-environmental, or even felt negative emotions when they realized that they were forced to bear a cost. In the past, most studies took it for granted that people voluntarily take specific pro-environmental behaviour [45] or apply the conclusion of positive PEB spillover to specific management implication without considering autonomy. However, if people felt forced to do some pro-environmental behaviour, their psychological process may change subtly. Due to the particularity of autonomy, it is difficult to be effectively manipulated in experimental research. As this study used the questionnaire survey method, we can identify a portion of samples who involuntarily bear the recycling cost through measurement and expanded the existing understanding of costliness on PEB spillover effect. Secondly, this research focuses on recycling behaviour, one subdivision of PEB, and is relatively less concerned as the last step of consumer behaviour. In the study of van der Werff et al. [7], the initial behaviour includes eight PEBs, and only one of which is related to recycling action. In our questionnaire, four items were used to measure individuals’ usual recycling efforts. In fact, filling the questionnaire is also a reminder of past PEB, which has been proved to promote other PEB [6,7]. As an important complement, our study shows that a single type of PEB, such as recycling behaviour, also has a signalling effect on environmental self-identity. Thirdly, different from using ecological product purchase intention as the outcome variable as in van der Werff et al. [7], the dependent variable of this study was a series of other PEBs, thus the research conclusion was more general.

In the past, many studies on the negative effect of behaviour spillover had a common feature, that was, the initial prosocial behaviour implemented by individuals was costless, and then the decline of subsequent prosocial behaviour was observed. For example, participants were asked to imagine engaging in community service [59], imagine purchasing green food [60], imagine engaging in the pro-environmental program [61], and writing positive stories about themselves [62]. Costless behaviour will not improve a person’s prosocial identity, thus it will lead to a moral licensing effect, while costly behaviour changes a person’s prosocial identity and leads to subsequent behaviour in line with the prosocial identity [45]. However, according to the result from this study, autonomy is also a factor that cannot be ignored. Take the study of Mazar and Zhong [60] for an example. Participants had no choice but to choose green products and then observed the negative spillover effect. This study shows that when a person cannot choose freely to implement pro-environmental actions, the PEB may lose the signalling function related to environmental self-identity.

Our research results show that in order to advance pro-environmental activities, publicity activities should pay attention to the pro-environmental actions that people have taken. For example, publicity activities can emphasize that people voluntarily participate in a series of common recycling behaviour, such as sorting garbage. Residents can be reminded by distributing leaflets or posting messages on the community bulletin board: have you sorted waste and recycled today? I recycle, I am proud! Only when a pro-environmental action has a strong signalling function can it enhance individual’s environmental self-identity and cause more PEBs. If the publicity campaign emphasizes a simple environmental action, it may not be enough. Therefore, publicity activities should focus on actions with strong signal characteristics. For example, the government can emphasize the high-cost recycling behaviour implemented by consumers in the past through customized information, because this may increase the signalling strength of the behaviour. China is promoting the compulsory classification and recycling of waste. In this process, special attention should be paid to emphasizing the autonomy of individuals to implement pro-environmental behaviour or weakening the feeling of being forced to recycling. For example, posting a banner with a thank-you message near the recycle bin: you’ve worked hard for recycling! The community appreciates your proactive efforts!

## 5. Conclusions

The study examined the potential influences of recycling efforts on subsequent pro-environmental behaviour. The results indicate that recycling efforts have a positive association with following pro-environmental behaviour through the mechanism of feelings of pride and environmental self-identity. We found that only when people autonomously perform costly recycling behaviour, the signalling strength of previous recycling efforts can promote environmental self-identity to the greatest extent. On the contrary, the high costliness weakens the signalling strength of previous recycling efforts through producing negative emotions.

Behaviour spillover effect has been extensively studied in many domains, such as prosocial behaviour, health behaviour, and pro-environmental behaviour. Scholars have reached consensus on some key issues, such as the moral licensing mechanism to explain negative spillover, the crucial role of identity in behaviour consistency, and the moderating role of behaviour difficulty, et al. Along with these advances, a holistic framework for unifying all findings is put on the agenda [50,63]. However, in the process of constructing such a theoretical building, the academic community ignores an important factor, i.e., the autonomy of behaviour, which is the focus of this study. In the research of behaviour spillover, most studies default that individuals voluntarily take specific behaviour. Although a few studies have pointed out the importance of whether or not individuals voluntarily take PEB, there is no empirical study on the key role of autonomy in behaviour spillover effect. In a sense, our study fills this research gap, which has obvious value for further research and the construction of the whole theoretical system.

This study also inevitably has some limitations. For example, there may be methodological deficiencies in using a single subjective questionnaire survey method and cross-section data. Besides, self-reports might introduce self-serving bias. For further research, it is necessary to combine behavioural experiment method and questionnaire survey method to obtain more reliable conclusions. Secondly, this study actually measures the intentions of future pro-environmental behaviour, which does not necessarily reflect the real behaviour of consumers. It is necessary to take field experiments or use second-hand data to verify the results of this study.

## Figures and Tables

**Table 1:** Constructs and measurement.

Construct	Item	Factor Loading	Cronbach’s α
RE	Q1	0.866	0.899
	Q2	0.854	
	Q3	0.812	
	Q4	0.848	
ESI	Q1	0.926	0.942
	Q2	0.88	
	Q3	0.919	
	Q4	0.85	
	Q5	0.822	
FoP	Q1	0.982	0.972
	Q2	0.976	
CoR	Q1	0.881	0.828
	Q2	0.891	
	Q3	0.889	
	Q4	0.886	
PEB	Q1	0.816	0.892
	Q2	0.789	
	Q3	0.788	
	Q4	0.856	
	Q5	0.866	
	Q6	0.725	
	Q7	0.842	
	Q8	0.818	
	Q9	0.768	
Autonomy	Q1	0.878	0.935
	Q2	0.912	
	Q3	0.856	
	Q4	0.912	
	Q5	0.918	
	Q6	0.845	
	Q7	0.871	
	KMO	0.961	
	Bartlett	0	

Note: RE = recycling efforts; ESI = environmental self-identity; FoP = feelings of pride; CoR = costliness of Recycling; PEB = pro-environmental behaviour.

**Table 2:** Descriptive statistics and reliability of measurement.

	M	SD	AVE	CR	1	2	3	4	5
1 RE	4.385	1.352	0.745	0.928					
2 CoR	4.282	1.428	0.782	0.921	0.321 **				
3 FoP	5.124	1.492	0.871	0.976	0.481 **	0.282 **			
4 ESI	5.562	1.193	0.782	0.952	0.422 **	0.132 *	0.613 **		
5 PEB	5.260	1.162	0.641	0.908	0.452 **	0.118	0.530 **	0.618 **	
6 Autonomy	5.186	1.484	0.612	0.872	0.512 **	0.140 *	0.478 **	0.552 **	0.780 **

Note: RE = recycling efforts; ESI = environmental self-identity; FoP = feelings of pride; CoR = costliness of Recycling; PEB = pro-environmental behaviour. * *p* < 0.05, ** *p* < 0.01.

**Table 3:** Path analysis of SEM.

Hypothesis	β	R^2^	t	*p*
Recycling efforts→Feelings of pride	0.418	0.282	8.625	0.000
Recycling efforts→Environmental self-identity	0.216	0.419	3.162	0.012
Feelings of pride→Environmental self-identity	0.524	0.419	9.126	0.000
Environmental self-identity→PEB	0.448	0.326	5.188	0.000

## Data Availability

The data presented in this study are available on request from the corresponding author. The data are not publicly available due to privacy.

## Appendix A

**Table A1:** Constructs and measurement.

Construct	Item
Recycling efforts (adapted from [64,65])	I usually separate and dispose of all recyclable materials.
	I have high involvement in recycling activities.
	I tend to buy products which can be recycled in the future.
	I have high adherence levels to separating and disposing of recyclable materials.
Environmental self-identity (adapted from [63])	I think of myself as an environmentally-friendly individual.
	I think of myself as someone who is very concerned with environmental issues.
	I would be happy to be seen as having an environmentally-friendly lifestyle.
	I would want my family and friends to think of me as someone who is concerned about environmental issues.
	I think everyone should contribute to environmental protection.
Feelings of pride (adapted from [39])	I am proud of my recycling efforts.
	I feel good about my recycling efforts.
Costliness of recycling (adapted from [52])	I spend time on recycling.
	I paid mental efforts for recycling
	I paid extra money for recycling.
	Recycling was easy for me (Reverse scoring).
Pro-environmental behaviour (adapted from Chinese General Social Survey, CGSS)	Discuss environmental issues with relatives and friends.
	Bring own shopping basket (bag) when shopping.
	Reuse plastic bags.
	Actively pay attention to environmental issues and information reported in the media.
	Donate for environmental protection.
	Actively participate in environmental publicity and education activities organized by social organizations.
	Actively participate in environmental protection activities organized by non-governmental environmental protection groups.
	Conservation of trees or green space.
	Actively participate in complaints or appeals for solving environmental problems.
Autonomy	I was passive.
	I did not like it.
	I was not willing.
	I was forced.
	I was under external pressure.
	I had no choice.
	I felt bound.
